# Epigenetic Maintenance of Acquired Gene Expression Programs during Memory CD8 T Cell Homeostasis

**DOI:** 10.3389/fimmu.2018.00006

**Published:** 2018-01-18

**Authors:** Hossam A. Abdelsamed, Caitlin C. Zebley, Ben Youngblood

**Affiliations:** ^1^Department of Immunology, St. Jude Children’s Research Hospital, Memphis, TN, United States; ^2^Department of Oncology, St. Jude Children’s Research Hospital, Memphis, TN, United States

**Keywords:** epigenetic regulation, CD8 T cells, homeostasis, immunological memory, cytokines

## Abstract

Memory CD8 T cells have a unique ability to provide lifelong immunity against pathogens containing their cognate epitope. Because of their ability to provide lifelong protection, the generation of memory T cells is now a major focus for current vaccination or adoptive cell therapy approaches to treat chronic viral infections and cancer. It is now clear that maintenance of memory CD8 T cells occurs through a process of antigen-independent homeostatic proliferation, which is regulated in part by the gamma chain cytokines IL-7 and IL-15. Here, we will describe the role of these cytokines in the survival and self-renewal of memory CD8 T cells. Further, we will describe the role of epigenetics in the maintenance of acquired functions among memory CD8 T cells during homeostatic proliferation.

## Memory T-Cell Homeostasis

Much of the inspiration for investigating the mechanisms involved in T cell development and maintenance, which ultimately identified common gamma chain cytokines, was borne out of the pathophysiology observed in patients with severe combined immunodeficiency. The ensuing discovery of mutations in the common gamma chain (γ_c_) receptor and its downstream signaling molecules further served as a major impetus for investigating the mechanisms that govern T cell homeostasis (1–5). Building upon these findings, a series of *in vitro* and *in vivo* studies confirmed the importance of γ_c_ cytokine signaling in T cell homeostasis (6–16). Notably, Berard et al. showed that low concentrations of IL-15 could promote the survival of naïve and memory murine CD8 T cells in the presence of MHC, whereas higher concentrations of IL-15 were sufficient to stimulate antigen-independent proliferation of memory CD8 T cells (15). Similarly, Cho et al. showed that exposure to high concentrations of IL-15 in addition to IL-2 induced extensive proliferation among naïve and memory CD8 T cells (16). These studies served to illustrate the pivotal role γ_c_ cytokines play in homeostasis of naïve and memory CD8 T cells.

The relationship between IL-15 signaling and CD8 T cell maintenance was further explored using animal models lacking IL-15 or IL-15Rα. In the absence of IL-15 or IL-15Ra, there is a marked reduction in T cells expressing high levels of CD44, a surrogate marker commonly used to identify activated T cells (7, 9). Furthermore, blocking IL-2/IL-15Rβ signaling *in vivo* in WT mice inhibited memory CD8 T-cell homeostatic proliferation (8). Because these studies were performed largely using polyclonal memory T cells in unimmunized mice, several subsequent investigations were performed with antigen-specific memory T cells. Using the vesicular stomatitis virus (VSV) and lymphocytic choriomeningitis virus (LCMV) mouse infection models, these studies demonstrated that the effect of IL-15 on memory CD8 T cells indeed served to preserve a *bona fide* long-lived memory CD8 T cell (6, 11). During VSV infection, IL-15Rα- and IL-15-deficient mice generated virus-specific memory CD8 T cells, but those cells incorporated BrdU poorly and the quantity of antigen-specific T cells declined over time (11). Similarly, it was reported using the LCMV model of acute viral infection that virus-specific memory CD8 T cells were unable to undergo homeostatic proliferation in the absence of IL-15 (6). From these studies, it became evident that IL-15 and its receptor play an important role in generation and/or maintenance of memory CD8 T cells.

In addition to IL-15, analyses of T cell turnover under lymphopenic conditions identified several other γ_c_ cytokines as regulators of T cell homeostasis. Specifically, IL-7 was found to be necessary for self-renewal of naïve CD8 T cells adoptively transferred into a lymphopenic environment (10, 12, 13, 17). Most notably, Goldrath et al. elegantly demonstrated that proliferation of adoptively transferred naïve polyclonal CD8 T cells is severely impaired by blocking IL-7Ra. However, blocking IL-15 signal had no effect on cell division indicating that naïve CD8 T cell proliferation is largely dependent on IL-7 (17). The requirement of IL-7 signaling for naïve T cells homeostatic proliferation was also demonstrated in studies showing that naïve CD8 T cells exhibit diminished survival/maintenance capacity after anti-IL-7 treatment in IL-15 KO mice or when naïve T cells are transferred into IL-7-deficient mice (12, 13). In contrast, irradiation of WT or *IL-15*-KO mice was sufficient to enable adoptively transferred memory T cells to undergo homeostatic proliferation, while basal homeostasis of memory CD8 T cells in intact mice required IL-15 signaling (17). Similar results were observed by Tan et al. in that adoptive transfer of memory CD8 T cells into irradiated *IL-15*-KO mice and blocking both IL-7 and IL-7Rα severely reduces the proliferative capacity of memory CD8 T cells compared to that in *IL-15*-KO or *IL-7*-KO mice (14).

While conclusions from both studies are generally consistent, it should be noted that the Tan et al. study observed that some memory T cells had undergone proliferation among the IL-15-KO-irradiated mice (14). In contrast the Goldrath et al. study reported a significant impairment in memory T cell proliferation in IL-15-KO mice (17). The discrepancy between both studies likely stems from the sorting strategy performed by each study. Goldrath et al. defined memory CD8 T cells as CD44hi CD122hi, while the Tan et al. study used a broader definition for T cell memory by isolating the total pool of CD44hi T cells, which include both CD122hi and CD122lo T cells. This discrepancy was later resolved as investigators began to assess the requirement of TCR signaling in survival of both naïve and memory T cells. Several ground-breaking studies lead to the conclusion that continuous contact with self-MHC-I/peptide complexes was critical for homeostatic proliferation and long-term survival of naïve CD8 T cells (18–20) whereas antigen-experienced memory CD8 T cells do not require MHC-I contact for their survival (18, 21–23). Among these studies were experiments performed by Boyman et al. where they generated bone marrow chimeric mice with WT and MHC-I KO T cells and measured the quantity of CD122hi versus CD122lo T cell subsets among WT and KO cells. Importantly, they reported a striking decrease in the frequency of MHC-I KO CD122lo CD8 memory T cells whereas the CD122hi population underwent only a modest decrease (24). These results resolve the discrepancy between the Tan et al. and Goldrath et al. studies while reinforcing the concept that survival of long-lived CD122hi memory T cells occurs through an antigen-independent mechanism. Collectively, these works advanced our understanding of memory T cell homeostasis and the specific role homeostatic cytokines play in this process. In the following sections, we will discuss the source of these cytokines, their known downstream signaling events, and how these signaling events may permit memory T cells to maintain or modify acquired gene expression programs during antigen-independent homeostasis.

## IL-7 and IL-15 Expression and Signaling

**Figure 1:**
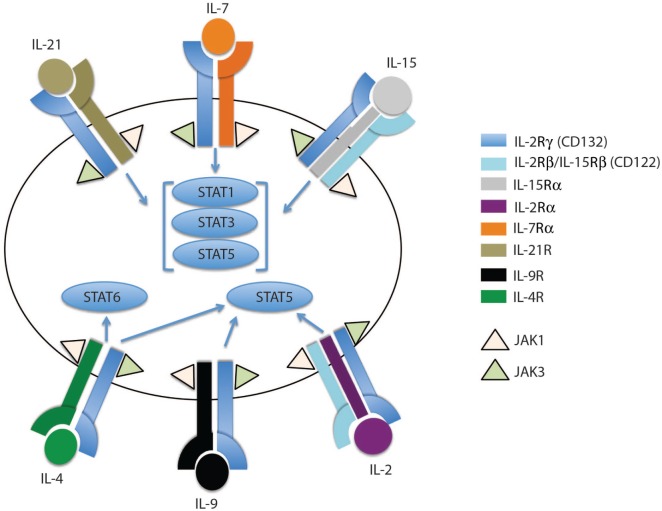
The family of common gamma chain cytokines and their receptors. The common gamma chain cytokine receptors are depicted in this cartoon showing IL-2Rγ (CD132) as the common subunit in all the receptors. Each receptor has its unique subunit that forms a heterodimer or heterotrimer receptor complex with the common gamma chain subunit. IL-4R, IL-7Rα, IL-9R, and IL-21R subunits dimerize with the common gamma chain subunit to form heterodimers that bind IL-4, IL-7, IL-9, and IL-21 cytokines, respectively. IL-2 and IL-15 receptors share two subunits, IL-2Rγ and IL-2Rβ, which trimerize with IL-2Rα or IL-15Rα to form the IL-2 or IL-15 receptor complex, respectively. The binding of each cytokine to its receptor complex results in phosphorylation of JAK1 and JAK3. The activated JAKs activate different STAT members, which then migrate to the nucleus to induce or inhibit expression of specific target genes.

IL-7 and IL-15 belong to the family of four alpha-helix bundle cytokines, including IL-2, IL-4, IL-9, and IL-21. The binding of IL-7 and IL-15 to their respective receptors activates several signaling pathways, including JAK/STAT and MAPK/PI3K-AKT, which results in the survival and proliferation of CD8 T cells. These cytokines are secreted by a wide spectrum of hematopoietic and non-hematopoietic cells and they bind to a multi-meric receptor complex sharing the common gamma chain receptor (γ_c_) (25, 26) (Figure 1). For instance, IL-7 is expressed in tissues, such as bone marrow, liver, and thymus, while IL-15 is found in bone marrow stromal cells, fibroblasts, kidney, skin, astrocytes, microglia, intestine, thymus, and retina (27–31). Further, under conditions of innate microbial triggers, antigen-presenting cells such as macrophages and dendritic cells become activated and express IL-15 mRNA (31, 32). Although initially IL-15 was not detected in T cells, Neely et al. and Thurkow et al. reported the expression of IL-15 protein by normal T cells and synovial tissue T cells from patients with rheumatoid arthritis (31, 33, 34). The widespread expression of IL-7 and IL-15 further highlights the importance of these cytokines in preserving protective immunological memory among tissues where antigen reencounter may occur again in the future. This point is further illustrated by recent discoveries in the area of memory T cell tissue residency. The Masoput lab, which has been pioneers in this field, have recently reported that the tissue-resident subset of memory T cells (Trm), which protect from reinfection in non-lymphoid tissues, are dependent on IL-15 for homeostasis (35).

**Figure 2:**
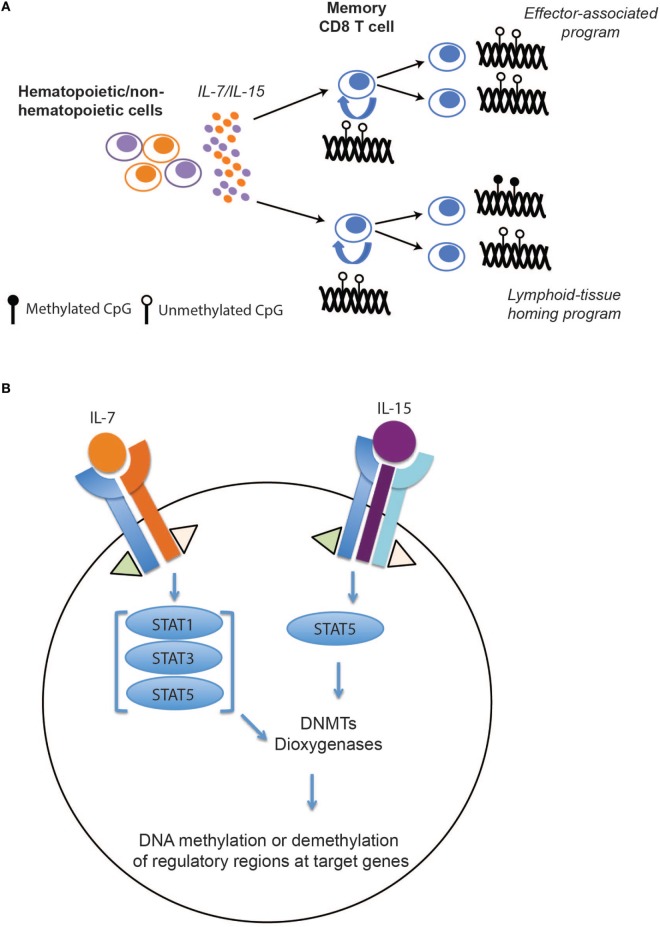
The effect of IL-7/15 on memory CD8 T cell epigenetic programs. **(A)** IL-7/15-mediated signaling and epigenetic propagation during homeostatic proliferation. IL-7 and IL-15 are expressed by hematopoietic and non-hematopoietic cells resulting in survival and proliferation of memory CD8 T cells. During memory CD8 T cell homeostatic proliferation, effector-associated programs, including IFNg, are maintained over several rounds of cell division while other programs such as CCR7 remain plastic during cell division. **(B)** Hypothetical model for selective modification of epigenetic programs during memory T cell self-renewal. Following IL-7 and IL-15 binding to their perspective receptors, activated JAK1 and JAK3 signaling proteins phosphorylate different members of STAT family. Activated STATs may induce or inhibit expression of key epigenetic enzymes, including DNA methyl transferases (DNMTs) and dioxygeneases, that result in *de novo* DNA methylation, maintenance, or demethylation of regulatory regions at target genes.

In general, gamma chain cytokines exert their effect through binding to cell surface or soluble receptors, activating downstream cell signaling and consequently transcription factors which regulate a myriad of cell processes including survival and proliferation. IL-7 and IL-15, as well as other members of this cytokine family, bind to a multi-meric receptor complex sharing the common γ_c_ receptor subunit CD132. The IL-15 receptor complex shares the β-chain (IL-2/IL-15Rb, CD122) with the IL-2 receptor, but it has a unique receptor subunit, IL-15Rα. Both IL-15Rα and Rβ subunits are preferentially expressed by memory CD8 T cells compared to their naïve counterparts (11, 14, 17, 36), potentially explaining why memory CD8 T cells are more responsive to IL-15 cytokine levels compared to naïve T cells. The effects of IL-15 are mainly achieved through a *trans*-presentation process, but have been shown to also occur, to a lesser extent, through *cis*-presentation (32). In the cell contact *trans*-presentation scenario, IL-15 binds to the IL-15Ra chain with high affinity (*K*_d_ ~ 10^−11^ M) and subsequently *trans*-presents this membrane-bound complex to cells expressing IL-15Rb-γ_c_ (37). Once IL-15–IL-15Ra forms a trimeric complex with IL-15Rb-γ_c_, the JAK–STAT pathway is activated, i.e., JAK1 and JAK3 are phosphorylated and subsequently recruit STAT3 and STAT5, respectively (Figures 1 and 2B). Consequently, phosphorylated STATs translocate to the nucleus, thereby promoting transcription of mitogenic and antiapoptotic genes (e.g., *BCL-2, MYC, FOS*, and *JUN*) and limit the expression of proapoptotic proteins, such as BIM and PUMA (38–40).

Complementing the IL-15 response, IL-7-receptor signaling activates a number of genes involved in survival and proliferation, such as the Bcl-2 family members, *BCL-XL, c-MYC*, and *D-cyclins*, as well as inhibits proapoptotic genes (Bad and Bax) (41, 42). Similar to IL-15, the IL-7 heterodimer has its own specific receptor, IL-7Ra (CD127), which is expressed by both naïve and memory CD8 T cells albeit the memory compartment showed higher surface expression compared to naïve counterparts (36). The investigations that lead to the identification of downstream signal targets of IL-7 and IL-15 highlight the complex nature and crucial role this signaling cascade plays in survival and proliferation of T cells. While it is now well established that maintenance of poised effector potential among memory CD8 T cells during homeostasis is important for host protection, an active area of investigation remains in determining how these acquired traits are preserved during cytokine-mediated proliferation. Notably, we have reported memory T cell subset inter-conversion, suggesting that these cytokines mediate plasticity in cell fates (43, 44). Since epigenetic mechanisms play an important role in cell fate decisions and differentiation, many groups are now examining the link between memory T cell differentiation, homeostasis, and stability/plasticity of epigenetic programs.

## Epigenetic Reprogramming of T Cells During Effector and Memory Differentiation

The highly regulated process of gene expression during cellular differentiation involves a myriad of epigenetic modifications, including histone modifications and DNA methylation, that mediate changes in chromatin accessibility at gene-regulatory regions that can instill the cell with a long-lived fate (45, 46). Histone acetylation decreases the positive charge of the nucleosome, consequently reducing the affinity of histone binding to negatively charged DNA and promoting transcription (47). Additionally, an assorted combination of methylated lysines among the histones can either promote or repress transcription. For instance, trimethylation of lysine 4 (H3K4me3) is mainly associated with gene expression and is enriched in promoters of actively transcribed genes. Conversely, methylation of lysine 27 (H3K27me3) is associated with transcription inhibition (48–50). In addition to histone modifications, DNA methylation is a well-established epigenetic modification that is generally associated with transcriptional repression. Methylation of DNA results in steric hindrance of transcriptional-activators and/or recruitment of transcriptionally repressive methyl-binding proteins.

Inspired by the findings that epigenetic programs function to reinforce cell fate decisions during early development, several investigators studying memory T cell differentiation have sought to determine if epigenetic modifications serve as a mechanism for maintaining acquired effector-associated functions during memory T cell homeostasis. Using both *in vivo* and *in vitro* models, several labs have demonstrated that the *Ifng* promoter in naïve CD8 T cell is heavily methylated and marked by H3K27me3-repressive histone modifications. However, the activation of naïve CD8 T cells *in vitro* or *in vivo* leads to rapid DNA demethylation, removal of H3K27me3, and deposition of permissive H3K9Ac and H3K4me3 marks (51–53). Similar findings have been reported for the proximal promoter region of granzyme B (*GzmB*), where the *GzmB* promoter becomes susceptible to nuclease activity after *in vitro* stimulation (54). In succession with these above-described loci-specific studies, recent genome-wide approaches have been undertaken to more broadly examine the epigenetic reprogramming (DNA methylation and histone modifications) that occur during the development of a naïve T cells into effector and memory CD8 T cells. In a study performed by Araki et al. the authors performed a genome-wide assessment of H3K4me3 and H3K27me3 marks in human polyclonal naïve and memory CD8 T cells and identified different classes of transcription patterns associated with the two histone marks. First, H3K4me3 marks were associated with actively transcribed genes. Second, H3K27me3 marks were associated with repressed genes and finally a bivalent mark was associated with genes, including many effector-associated loci that are potentially poised for expression (55). To further explore the degree of epigenetic reprogramming associated with effector differentiation, Scharer et al. recently generated a global snapshot of the methylation status of naïve and effector CD8 T cell genomes following LCMV infection in mice. The authors identified approximately 650,000 differentially methylated regions between the two populations using a MeDIP-Seq approach (56). Together, the results from loci-specific and genome-wide studies provide evidence for significant plasticity of histone modifications and DNA methylation in response to TCR stimulation in CD8 T cells. Specifically, these studies document the epigenetic reprogramming of effector-associated loci. Importantly, these results also provide a potential mechanism for explaining the long-lived poised effector status of memory CD8 T cells and have prompted investigation into the acquisition and stability of these programs in self-renewing memory T cells.

The discovery that T cell effector differentiation is coupled to the acquisition of transcriptionally permissive epigenetic modifications at effector loci has further fueled the heavily debated issue centered on whether memory T cell differentiation progresses through an effector stage whether or not. We recently addressed this issue using mouse and human models of acute viral infection model. In one of our recently published studies examining the development of virus-specific mouse memory CD8 T cells, we provide evidence that memory T cells arise from a subset of effector T cells (Memory Precursor) that retain an epigenetic signature of an effector response (57). Along the same lines, Akondy et al. addressed the question of memory T cell origin by *in vivo* labeling rapidly dividing cells with deuterium during the effector stage of the immune response to yellow fever vaccination. YFV-vaccinated individuals consumed “heavy” water during the first 2 weeks of the immune response to the vaccine, and then deuterium incorporation among the virus-specific T cells was measured longitudinally. Strikingly, virus (YFV)-specific memory T cells remained fully labeled with deuterium more than a year post vaccination. These data demonstrate that human memory CD8 T cells are derived from a population of cells undergoing a burst in cell proliferation during the effector stage of an immune response. Moreover, the YFV-specific memory T cells retained an epigenetic fingerprint similar to the YFV-specific effector CD8 T cells (58). These studies provide further evidence that memory T cell differentiation is coupled to epigenetic modifications of effector-associated loci.

In addition to the studies focused on DNA methylation described above, Russ et al. have examined changes in histone modification during effector and memory T cell differentiation using the influenza A mouse model of acute viral infection. They observed that many of the effector-associated loci that acquired permissive histone modifications in effector CD8 T cells also acquired these histone modifications in memory CD8 T cells. Notably the promoter and gene bodies of several effector-associated molecules (i.e. *IFN*γ, *GzmB*, and *GzmK*) among the memory CD8 T cells acquired a transcriptionally permissive state (59). Similar to Russ et al.’s study, Crompton et al. performed a genome-wide assessment of histone modifications, but using *in vitro-*generated murine memory CD8 T cells subsets (Tem, Tcm, and Tscm cells). They observed that regulatory regions of effector loci (*GzmB, IFN*γ, and *Prf*) were highly enriched with permissive histone modifications in both Tem-like and Tcm-like cells (60). Taken together, these data reinforced the idea that effector-associated loci acquire transcriptionally permissive histone modifications during memory CD8 T cell differentiation.

While these mouse and human studies leave little doubt that memory T cell differentiation is associated with epigenetic reprogramming of effector-associated loci, the question of whether or not these programs are stably propagated during memory CD8 T cell homeostatic proliferation remained unanswered. To address this question, we recently defined the DNA methylation programs among human naïve and memory CD8 T cell subsets and asked whether the acquired epigenetic states of memory T cells were maintained during antigen-independent self-renewal. Naïve and memory CD8 T cell subsets (Tem, Tcm, and Tscm) were freshly isolated from healthy individuals and the methylation profile of these populations were determined at a nucleotide level of resolution by performing whole-genome bisulfite sequencing. Importantly, we observed that many effector-associated loci were methylated in naïve CD8 T cells, but were unmethylated in the long-lived Tcm and stem-cell like (Tscm) memory CD8 T cell subsets (e.g., IFNg and GzmK) (44). Having identified DNA methylation programs that were coupled to the poised effector state of long-lived human memory CD8 T cells, we next asked if these programs persisted during gamma chain cytokine-mediated proliferation. Indeed, memory CD8 T cells, including the longest lived subset Tscm, maintained their unmethylated state despite having undergone several rounds of cytokine-mediated cell division (Figure 2A). Additionally, proliferation alone was not sufficient for inducing a demethylated state among the naïve CD8 T cells. In contrast to the effector-associated loci, the lymphoid-tissue homing marker CCR7 loci underwent changes in methylation status that correlated with phenotypic changes in the cell (Figure 2A), indicating that stability of epigenetic programs among self-renewing memory CD8 T cells was a loci-specific process.

Taking advantage of a recently developed clinical protocol whereby CD45RAneg CD8 T cells from a haploidentical donor are adoptively transferred into lymphopenic bone marrow transplant patients (61), we were able to extend our observations made from *in vitro* studies by examining the methylation status of effector-associated methylation programs in memory T cells undergoing *in vivo* antigen-independent proliferation. Quite strikingly, the demethylation status of the effector programs (IFNg and Prf1) was remarkably stable, as the donor memory CD8 T cells recovered after several months of *in vivo* homeostasis among lymphodepleted recipients retained demethylated effector-associated loci. Collectively, these studies highlight epigenetic modifications as a mechanism for preserving acquired gene expression programs among memory CD8 T cell during antigen-independent homeostasis.

## Conclusion and Future Directions

The collective studies described above broadly highlight the critical role common gamma chain cytokines play in naïve and memory CD8 T cell homeostasis. While our review focused primarily on IL-7 and IL-15, it should be noted that other family members of the gamma chain cytokines, including IL-2 and IL-21, play an important role in naïve and memory CD8 T cell homeostasis (62–64).

Because of their crucial role in preserving T cell immunity, a wide range of hematopoietic and non-hematopoietic cells expresses these cytokines. They exert their function through complex signaling pathways, including JAK/STAT and PI3K/MAPK, and regulate the expression of cell cycle, apoptotic, and antiapoptotic genes. However, how these signaling events regulate DNA methyl transferases (DNMTs) and dioxygenases (TETs) specificity—enzymes regulating addition, maintenance, and DNA demethylation—remains to be explored (Figure 2B). A recent study from our lab suggests that the methylation status of CpGs at several genes involved in memory T cell subset specification are sensitive to IL-7 and IL-15 signaling (44). While little is known regarding the specificity determinants for site-specific DNA demethylation and *de novo* methylation during T cell differentiation or homeostasis, recent advances in understanding the relationship between DNA methylation reprograming and transcription factor binding in other developmental systems may provide insight (65, 66). Building upon the observations that DNMT specificity can be regulated by transcription factor localization, a particular focus on assessing the relationship between *de novo* methylation and activation and localization of transcription factors down stream of the common gamma chain signaling cascade may be warranted. Conversely, recent studies examining the mechanisms regulating DNA demethylation have revealed cell division-dependent mechanisms that involve hydroxylation of methylated cytosines by TET enzymes. Hyrdroxylation in turn inhibits binding of DNMT1, the methyltransferase responsible for maintenance of DNA methylation, which consequently blocks propagation of DNA methylation programs to the newly synthesized daughter strand during DNA replication (67, 68). Thus, one can envision gamma chain cytokines modifying TET activity and promoting cell division-dependent demethylation during memory T cell homeostasis.

Further investigation into such cytokine-mediated changes in epigenetic programs among memory T cells will be important to guide efforts that seek to promote and maintain memory T cell subsets with desired functions. In addition to further mapping the upstream signaling events that promote changes in T cell epigenetic states, in depth investigation is needed into the role of epigenetics in T cell function during homeostasis (Figure 2B). While we have recently demonstrated that Dnmt3A-mediated *de novo* DNA methylation programs are causal in establishing T cell exhaustion that limit the efficacy of immune checkpoint blockade therapies (69), further understanding of the other epigenetic mechanisms involved in the development of T cell exhaustion will likely provide additional targets for therapeutic intervention. Thus, our ability to direct or skew the epigenetic state of memory T cells awaits further discoveries that will likely have major implications for current and future immunotherapeutic approaches that employ T cells in the treatment of chronic infections or cancer.

## Author Contributions

All authors contributed to writing and editing the manuscript.

## Conflict of Interest Statement

The authors declare that the research was conducted in the absence of any commercial or financial relationships that could be construed as a potential conflict of interest. The reviewer TE and handling editor declared their shared affiliation.
